# Water, sanitation and hygiene (WASH) index for primary healthcare facilities: Towards achieving WASH security

**DOI:** 10.1016/j.heliyon.2024.e35548

**Published:** 2024-08-02

**Authors:** Enovwo E. Odjegba, Abayomi O. Bankole, Adebayo Sadiq, Barakat O. Layi-Adigun, Abayomi M. Adebimpe, Mariam O. Kosemani, Emmanuel B. Ojo, Mustapha A. Adewuyi

**Affiliations:** a Federal University of Agriculture, Abeokuta, (FUNAAB), P.M.B 2240, Ogun State, Nigeria; b Kent State University, Kent, OH, USA; c São Paulo State University, Faculty of Engineering, Bauru Campus, São Paulo, Brazil

**Keywords:** Primary healthcare facilities, WASH, LMICs, Service ladders, Indicators

## Abstract

This study focused on designing a WASH Index for assessing the status of WASH in Primary Healthcare Facilities (PHCs) especially for low- and middle-income countries. To assess the effectiveness of the WASH Index in evaluating the WASH in PHCs, PHCs were selected from 70 Local Government Areas (LGA) across 3 Southwestern States in Nigeria. The WASH index comprises of the five Joint Monitoring Programme service ladders as outlined in the World Health Organization Global Baseline Report for monitoring basic WASH services in health care facilities: water, sanitation, hygiene, waste management and environmental cleaning. The 5 service elements (termed as components) assessed were based on 10 indicators and 30 sub-indicators. The results of the WASH Index of the PHCs were compared statistically on LGA and State basis with emphasis on the status of WASH facilities. The study concludes that the result would further provide baseline information on the status of WASH in PHCs in the selected States in the quest to achieve the Sustainable Development Goals (SDGs). This study recommends that the WASH Index could be adopted an assessment tool for evaluating WASH in PHCs in other to ensure communication of results to policy makers and other relevant stakeholders, for effective monitoring healthcare facilities.

## Highlights

• WASH Index was developed to assess status of Primary Health Care facilities.
• The index is useful for measuring service ladders in Primary Health Care facilities.
• The index is result comprehensible for relevant stakeholders and policymakers.
• The index promote comprehensive monitoring of service level indicators in LMICs.

## Graphical abstract

**Figure:**
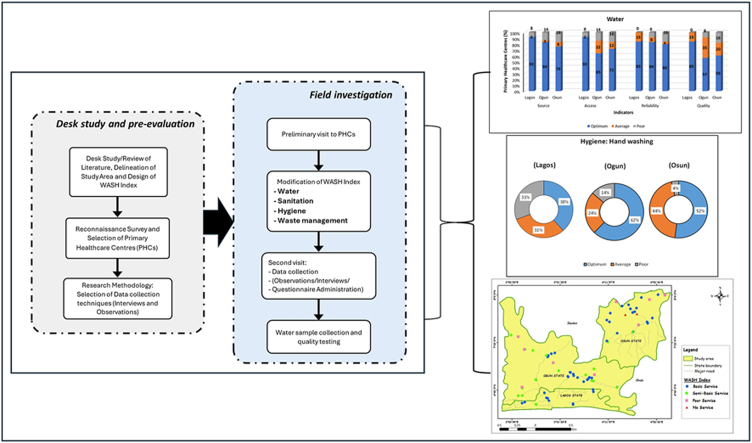

## 1 Introduction

Water, Sanitation and Hygiene (WASH) are vital components of effective service delivery in healthcare facilities. WHO (2020) reports that 25 % of healthcare facilities have no basic water services, about 712 million people lacking access to water when they visit healthcare facilities. The report continues that with respect to sanitation, 10 % of healthcare facilities worldwide have no sanitation services, adequate hand cleaning facilities at points of care, and improperly segregated waste. Provision of adequate WASH facilities in healthcare facilities is vital in preventing the incidence and spread of healthcare acquired infections [1,2], and poor sanitation have been linked to several bacteria and diseases [3,4]. Improved WASH access of healthcare facilities in low- and middle-income countries (LMICs) have significant effect on patient's satisfaction and health outcomes [5]. The state of the healthcare facility environment otherwise described as the physical environment by Ref. [6] also influences the service delivery of hospital staff. Inadequate WASH facilities have heightened job frustrations that maybe transferred to patients.

Access to healthcare services; especially Primary Healthcare Centres (PHCs) in LMICs is impeded in part, by inadequate facilities, poor patient–staff ratios and limited or total absence of WASH and healthcare waste management facilities [7,8]. In Nigeria, PHCs, intended to be the initial medical point for accessible basic healthcare service delivery prior to moving to hospitals sadly falls short of this fundamental function. The PHCs serve a key part of the general population, however, basic and cost-effective services are still absent in a significant sections, especially in rural communities [[9], [10], [11]]. It is observed that some PHCs had dilapidated structures, poor sanitation, inadequate staffing and paucity of medical equipment, a contrast to the technical guide by the National Primary Health Care Development Agency (NPHCDA) for PHCs in Nigeria [12].

The NPHCDA technical guide outlines the basic requirements for ensuring a clean and healthy PHC in line with national developmental objectives and international best practices [7]. In the quest to achieve Sustainable Development Goals (SDGs), Nigeria's Federal Ministry of Health through the NPHCDA introduced the Primary Health Care Under One Roof (PHCUOR) initiative as a policy in 2010 [13]. PHCUOR also called Integrated PHC Governance is targeted at strengthening Nigeria's health system using the principle of “One Management, One Plan and One Monitoring and Evaluation (M&E)”. NPHCDA (2015) states that PHCUOR integrates PHC structures/programmes under the State Primary Health Care Development Agency or Board (SPHCDA/B).

The WASH sector is described as complex, involving multiple actors and factors and multiple data [14]. Better use of acquired data is therefore important for the goal of WASH to be achieved [15]. However, there are limitations with regards to data on access to WASH facilities in LMICs especially for rural healthcare facilities [2,16]. This study believes that the use of an index approach in assessing the status of WASH in healthcare facilities, especially PHCs could demystify the complexities in WASH. Several indices and models have been designed for measuring WASH status at individual, household, and country levels (Table 1). The highlighted literatures all focus on WASH in relation to country, state, and household levels; sometimes specifying WASH as it relates to individuals.

**Table 1:** Summary of some selected WASH related indices and models.

S/N	Indices/Model	References	Description
1.	Water Poverty Index (WPI)	[17]	WPI was developed as a tool for measuring household and community level water stress.
2.	Hygiene Index (HI)	[18]	HI was generated from four specific indices: drinking water index, food index, personal hygiene index, and domestic household hygiene. The objective of the study was to access the ability of the index to predict early childhood diarrhoea.
3.	Water and Sanitation Sustainability Index (WASSI)	[19]	WASSI is a sustainability index for assessing water and sanitation management systems.
4.	Municipal Basic Sanitation Index (MBSI)	[20]	MBSI is a tool designed to enable municipal sanitation monitoring in Sao Paulo, Brazil
5.	Peri-Urban Healthy Toilet Index (PUHTI)	[21]	PUHTI was developed from the Healthy Sanitation Framework. PUHTI was used to measure on-site peri-urban sanitation quality in Lusaka, Zambia
6.	Sanitation Index	[22]	The index measures sanitation coverage by recognizing the public health and environmental impacts of different sanitation technologies
7.	WASH Poverty Index	[23]	It is a multidimensional tool, linking poverty and WASH related issues.
8.	WASH Performance Index	[24]	The index compares the performance of countries at realizing universal WASH using water access, water equity, sanitation access, and sanitation equity components.
9.	IBM-WASH Model	[25]	It is an Integrated Behavioral Model for Water, Sanitation and Hygiene in the form of a matrix. The model uses three intersecting dimensions that influences WASH behaviours: the contextual dimension, psychosocial dimension and technological dimension.
10.	Composite Index	[26]	Used composite index (drinking water index, personal hygiene index and household hygiene index) to assess personal hygiene and sanitation amongst adolescents' girls and their households in Maharashtra, India
11.	Water Supply Systems Sustainability Index (WSSI)	[27]	Assessed the sustainability of water supply systems using five factors: access, quality, reliability, cost and management. The Index results in a composite score was combined with the results of sanitary risk assessment score of the water supply systems

The Water Poverty Index (WPI) was developed by Ref. [17]. Like the WASH Index developed in this study, the WPI is a composite tool that sought to link indicators of water and human welfare to the degree of the impact of water scarcity on human populations. The WPI centred around five [4] components: resources, access, capacity, use and environment, which cuts across water and environment which is similar to the components of the WASH Index. The Hygiene (HI) is a combination of four [3] specific indices: drinking water index, food index, personal hygiene index, and domestic household hygiene [18]. The HI can be considered as a composite which assessed the ability of the index to predict early childhood diarrhoea. The term hygiene, closely linked with water, is also a key component if WASH related studies. The HI is related to the sanitation and hygiene components of the WASH Index. Water and Sanitation Sustainability Index (WASSI) assesses water and sanitation management in Argentina [19]. WASSI comprised three [2] sub-indices: ‘place’, ‘permanence’, and ‘persons’. ‘Place’ sub-index evaluates water availability, infrastructure and coverage, ‘permanence’ looks at access, planning and participation, while ‘persons’ assess water use, impact and water user satisfaction. WASSI broadly evaluates water and sanitation that are also components of WASH Index.

Municipal Basic Sanitation Index (MBSI) was designed by Ref. [20] as a tool to enable municipal sanitation monitoring in Sao Paulo, Brazil. The MBSI was conceived due to increased requirement of composite indicators for measuring the performance cities particularly for the generation of information for public administration. Interestingly, MBSI uses composite indicators like WASH Index and also considers effective generation of information for policy makers. The Peri-Urban Healthy Toilet Index (PUHTI) was developed by Ref. [21] from the Healthy Sanitation Framework (HSF). PUHTI was created for on-site evaluation of peri-urban sanitation quality in Lusaka, Zambia. The HSF highlighted five classifications of sanitation quality, which are hygiene, use, sustainability, desirability, and accessibility; WASH Index equally captures sanitation, hygiene and most importantly toilet facility adequacy. In addition, the Sanitation Index was designed in Sri-Lanka by Ref. [22] for measuring sanitation coverage by recognizing the public health and environmental impacts of different sanitation technologies. The index used indicator variables defined under two categories to create two sub-indices: latrine security and hygiene and treatment and disposal. Sanitation Index when compared with WASH Index, is similar in relation to toilet facility security and hygiene (captured under ‘Toilet facility cleanliness and state of structure’ in WASH Index).

The WASH Poverty Index (WASH PI) links poverty and WASH related issues [23], using Kenya as a case study. WASH PI was designed as an inter-disciplinary, multi-dimensional and WASH-focused approach. The WASH PI is a composite of three indices, the Water Poverty Index, Sanitation Poverty Index and the Hygiene Poverty Index. The WASH PI identifies deprivations in WASH services at the household level for better understanding of the link with poverty. While WASH Index did not capture poverty directly, The WASH PI is significantly related to WASH Index considering the incorporation of WASH components. WASH Performance Index [24] focused on comparing the performance of countries in realizing universal WASH based on water access, water equity, sanitation access, and sanitation equity components. Although the WASH Performance Index utilizes similar components as WASH Index, the WASH Performance Index views WASH on a global scale, while WASH Index is country specific.

The IBM-WASH Model as an Integrated Behavioral Model for Water, Sanitation and Hygiene in the form of a matrix [25]. The model consists of three intersecting dimensions that influences WASH behaviours: the contextual dimension, psychosocial dimension, and technological dimension. Although, IBM-WASH Model like WASH Index is an assessment tool for WASH evaluation, the model's primary focus is the provision of a practical tool to aid the understanding and assessment of multiple factors that affect WASH practices especially in areas with infrastructural limitations. Composite Index is a composite index involving three indices: drinking water index, personal hygiene index and household hygiene index [26]. The resulting composite index assesses personal hygiene and sanitation amongst adolescents' girls and their households in Maharashtra, India. While WASH Index is not gender specific, WASH Index relates directly to the Composite Index in hygiene and sanitation only. The Water Supply Systems Sustainability Index (WSSI) was designed to assess the sustainability of water supply systems using five factors: access, quality, reliability, cost, and management [27]. The Index results in a composite score was combined with the results of sanitary risk assessment score of the water supply systems. However, WSSI only captures ‘water’ component unlike the WASH Index that extends to sanitation and hygiene.

There are limited studies on indices and models accessing as it relates to WASH in healthcare facilities. WASH in healthcare facilities is more complex, basically due to the numerous contamination pathways and specifically, the infectious levels of the contamination. This study believes that one way to assess WASH in healthcare facilities and covey results in the most explicit form for policy makers and other relevant stakeholders to comprehend, is to present such results in the form of an index. The index approach simplifies complexity without losing the WASH focus [23]. Hence, a WASH Index that is centred on accessing the status of water, sanitation, and hygiene in healthcare facilities and providing composite results that is easy to understand in necessary.

This study is a pilot study to design a WASH Index to assess the status of WASH in Primary Healthcare Facilities. Previously [7], had used [28] Joint Monitoring Programme (JMP) service ladder to assess the status of WASH in PHCs by classifying the WASH facilities into: Basic service, Limited service and No service. It was observed that classifying WASH facilities into Basic, Limited service and No service may not sufficiently represent the levels of improvement in the status of WASH facilities in PHCs especially in LMICs [7]. For instance, with respect to sanitation component of the JMP service ladder [28], PHCs can only classify as Basic service if ‘Improved sanitation facilities are useable, with at least one toilet dedicated for staff, at least one sex-separated toilet with menstrual hygiene facilities, and at least one toilet accessible for people with limited mobility’. The study argued that possibilities exist of PHCs meeting dedicated/sex-separated toilet and limited mobility accessibility criteria but fall short of the availability of menstrual hygiene supplies (due to the high cost of menstrual hygiene supplies); thereby relegating such PHCs to Limited service. Also, only water, sanitation and hygiene components of the JMP service ladder was covered, excluding the waste management and environmental cleaning components [7].

In this study, five components of the JMP service ladder: water, sanitation, hygiene, waste management and environmental cleaning were adopted to design the WASH index for healthcare facilities. Authors believe that the goal of ensuring improved WASH facilities in healthcare facilities in general (not just PHCs) is necessary to achieve WASH security. WASH security is the process of guaranteeing the availability of WASH facilities, where and when needed, adequate with respect to stipulated standards for current and future use. WASH security is not limited to healthcare facilities but should cut across households, schools, workplace and other spheres of the environment.

In this paper, the methods used to design the WASH index for PHCs are discussed, and the approach used to compute the final index are also presented. A reflective discussion to highlight the implications of the WASH index is equally included and the paper concludes with relevant recommendations and suggestions for further research.

### 1.1 The study area

**Fig. 1:**
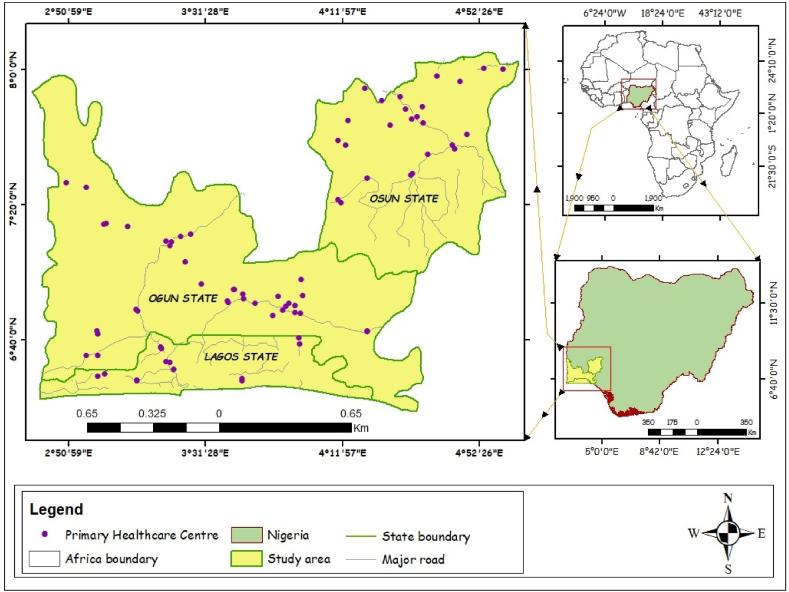
Map of study area.

The study covered 3 States in Southwest Nigeria: Lagos, Ogun and Osun State (Fig. 1). The States have a total of 70 Local Government Areas, Lagos [19], Ogun [19] and Osun [29]. The choice of study area is based on authors' familiarity with the terrain. Currently, at 2.6 % annual growth rate [29] and 2006 National Population Census, Lagos is the most populated of the 3 States (13,054,290 persons), followed by Ogun (5,373,160 persons) and Osun (4,894,438 persons) States. Since the commencement of PHCUOR, States through their respective SPHCDA/B are gradually implementing the guidelines summarized in 9 pillars, namely: Governance & Ownership, Legislation, Minimum Service Package, Repositioning, Systems Development, Operational Guidelines, Human Resources, Funding Sources & Structure and Office Setup. WASH components are captured in the 9th Pillar (Office Setup). The 9th Pillar requires PHCs’ office structure to be a building that accommodates all staff, have a source of clean water and functional toilet facilities among others [30]. It is reported that as Office Setup is currently the best performing of all the 9 pillars [13].

## 2 Methodology

**Fig. 2:**
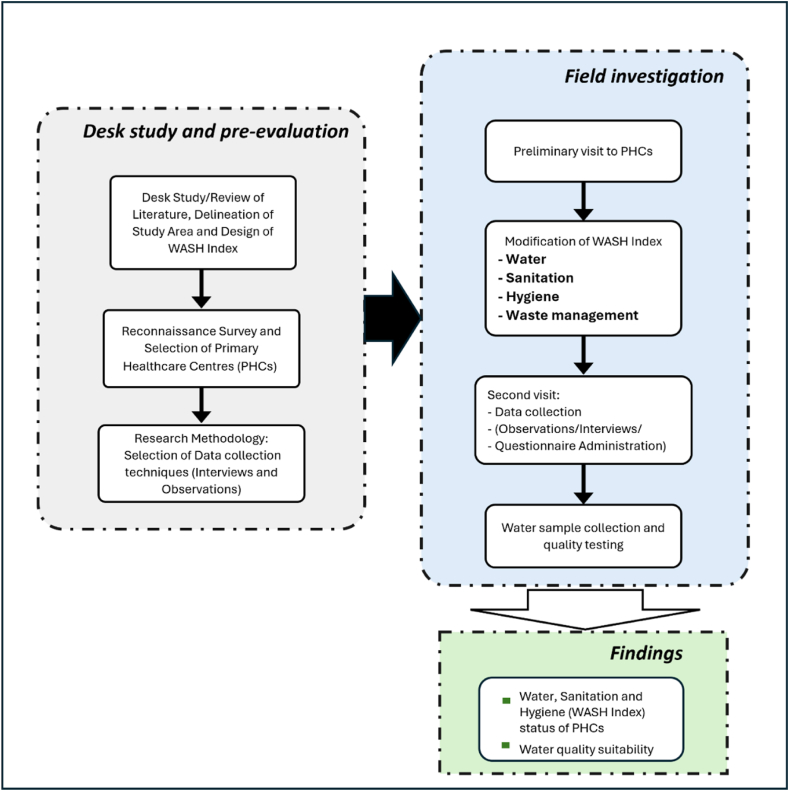
Research framework.

The conceptual framework of the study is presented in Fig. 2. The study commenced with a desk study that involved the review of relevant literature relating to WASH related indices, delineation of the study area and designing of the WASH index. The study was initially designed to cover all 70 LGAs across the 3 states with the selection of 2 PHCs each (totalling 140 PHCs), however, the entire LGAs could not be covered due to.

inaccessibility, functionality of PHCs and inadequate funding. Seventy-five [31] PHCs were assessed in the 3 States: Lagos [12], Ogun [32] and Osun [24]. The PHCs selected using the random sampling technique. The selected PHCs were assessed using designed WASH index. The WASH index in presented in Table 2. In addition, water samples were collected from water sources used by the PHCs and the samples were tested for pH, total dissolved solids (TDS), chloride, nitrate and *E. coli*.

**Table 2:** WASH index assessment sheet for primary healthcare centres (Adapted from Refs. [27,28]).

**Components**	**Indicators**	**Definition**	**Sub-indicators**	**Score** **Obtainable**	**Score** **Obtained**
**Water**	Source type	Source type describes the type of water source used in the Primary Healthcare Centre (NPHCDA)	A. On premises improved Source e.g. groundwater (protected hand-dug wells and boreholes)	2	
			B. On premises unimproved sources (hand-dug wells with no covers or lining)		
			C. Not on premises water source and reliance on sources away from PHC e.g. groundwater and/or surface water	0	
	Access	Access is defined with respect to the proximity of the water source to the point of use (NPHCDA)	A. On premises with building connection	2	
			B. On premises without connection	1	
			C. Water is sourced outside PHC premises	0	
	Reliability	Reliability is a description of how available water is in the water source, for instance, if the yield of water is influenced by seasons	A. Water is consistently available in adequate quantity	2	
			B. Quantity of water used influenced by distance of collection point to point of use (NPHCDA)^a^	1	
			C. Quantity of water used varies with yield or season	0	
	Quality	Quality describes the contamination level of water from the water source.	A. Source is protected	2	
			B. Source is protected but close to possible sources of contamination	1	
			C. Water source is unprotected	0	
**Sanitation**	Toilet Facility Condition	Toilet facility type covers the technology type (e.g. flush type/pit latrine and so on)	A. Flush/Mobile/Pit Latrine in exceptionally good condition	2	
			B. Flush/Mobile/Pit Latrine in slightly good condition	1	
			C. Flush/Mobile/Pit Latrine in bad condition and not useable/open defecation	0	
	Toilet facility cleanliness and state of structure	Toilet facility cleanliness and state of structure describes level of cleanliness and the state of the toilet facility structure	A. Toilet facility is clean, and structure is intact	2	
			B. Toilet facility structure is intact, but facility is dirty	1	
			C. Toilet facility is dirty, unkempt and structure is dilapidated	0	
	Toilet facility Adequacy	Toilet facility Adequacy is defined in terms of number and condition of toilet facility/facilities in the PHC	A. Toilet not shared/Gender demarcated/Menstrual and Hygiene Supplies available	2	
			B. Shared by Patients/Staff	1	
			C. No toilet	0	
**Hygiene**	Hand washing	Hygiene captures the hand washing component of the PHC.	A. Hand wash station with soap and water	2	
			B. Hand wash station present with water but without soap	1	
			C. Hand wash station present but not functioning/Hand wash station completely absent	0	
**Waste** **Management**	Waste Management and disposal	Waste management and disposal describes the kind of waste disposal practices employed in the PHC	A. Dumpster present and emptied regularly	2	
			B. Dumpster present and overflowing	1	
			C. Dumpster absent, waste disposed openly	0	
**Environmental** **Cleaning**	Environment (Surroundings)	Environmental cleaning defines the level of cleanliness of the PHC	A. PHC interior and surrounding is clean	2	
			B. PHC interior is clean but surrounding with grown bushes	1	
			C. PHC interior dirty and surrounding with grown bushes at the time of visit	0	
**WASH Index =** $\frac{Score\;Obtained}{Score\;Obtainable}\times 100$				20	

a NPHCDA: National Primary Health Care Development Agency; A = Optimum, B = Average, C = Poor.

### 2.1 WASH index computation

The WASH index is composed of indicators and sub-indicators that were designed to further aid assessment of each 5 WHO/UNICEF (2018) JMP service ladder components. The WASH Index was patterned after Water Supply Systems Sustainability Index (WSSI) from Ref. [27]. The indicators for Water component include source type (type of water source used by the PHC), access (defined with respect to proximity of the water source to point of use: the PHC), reliability (assesses how readily available water is from the water source), and quality (how protected the water source is from contamination).

For Sanitation and Hygiene, the indicators are toilet facility (covering the type, number and condition of toilet facility being used in the PHC) and hand washing (presence/functionality of a hand wash station) respectively. Waste management component indicator is waste management and disposal, which describes the kind of waste management and disposal practices employed in the PHC, while Environmental cleaning component indicator defines the level of cleanliness of the PHC. All indicators are further described using sub-indicators for appropriate scoring.

To effectively assess the 5 components, indicators were scored using the sub-indicators. Each indicator could score a maximum of 2 and minimum of zero. The sub-indicators were necessary to adequately categorize the indicator scores into three levels: Score of 2 (Optimum), Score of 1 (Average) and Score of zero (Poor). Hence the total maximum obtainable score for all 10 indicators is 20. The WASH index was determined using Equation (1) below:

(1) $$WASH\;Index=\frac{Score\;obtained}{Score\;obtainable}\;x\;100$$

The resulting WASH Index scores were classified as Basic Service (76–100 %), Semi-Basic Service (51–75 %), Poor Service (26–50 %) and No Service (0–25 %) as indicated in Table 3.

**Table 3:** WASH index classification and interpretation.

WASH Index Classification	Score Range	Description
Basic Service	76–100 %	Water source is accessible on premises and reliable with building connection and protected, toilet facility clean, gender/staff/patient demarcated and in good condition and hand wash station functional. Waste dumpster present and emptied regularly and environment clean. PHCs in the Basic Service category should not record score of one [33] for more than 4 indicators and should obtain at least a score of 1 in indicators within water, sanitation and hygiene component.
Semi-Basic Service	51–75 %	PHCs in the Semi-Basic Service category should record indicator scores between 10 and 15. Water source is accessible on premises and reliable without building connection and protected but close to possible contamination source, toilet facility clean, gender/staff/patient demarcated and in slightly good condition and hand wash station functional. Waste dumpster present and emptied regularly and environment clean.
Poor Service	26–50 %	Water source is not available on premises, external source is accessible, and distance does not limit quantity of water use, toilet facility is clean and in slightly good condition but shared by staff and patient; hand wash station present with water but no soap or vice versa. Waste dumpster present but overflowing and inside the PHC is clean but overgrown bushes outside.
No Service	0–25 %	No water source is on premises, water is taken from water sources (ground or surface) more than 500 m. Water availability is influenced by yield and season. Toilet facility is in bad condition/No toilet facility. Hand wash station is absent, waste is disposed openly, and environment is dirty.

The JMP service ladder classification (Basic service, Limited service and No service) was modified in this study as Basic service, Semi-Basic service, Average (Poor) Service and Poor (No) service in response to the observation of [7] regarding the representativeness of the levels of improvement in the status of WASH facilities in PHCs especially in LMICs as stated earlier.

Basic service indicates that the on-premises water source is accessible and reliable with building connection and protected, toilet facility clean, gender/staff/patient demarcated and in good condition and hand wash station functional. Also, waste dumpster present and emptied regularly and environment clean. In grading the sub-indicators, PHCs in the Basic Service category should not record a score of one [33] for more than 4 indicators. Semi-Basic Service implies that PHCs should record indicator scores between 10 and 15. Water source is on premises, accessible and reliable but without building connection and protected but close to possible contamination source. Likewise, toilet facility is clean, gender/staff/patient demarcated and in slightly good condition and hand wash station is functional. Waste dumpsters are present and emptied regularly and the environment is clean.

For PHCs in the Poor Service category, water source is not available on premises, source is accessible but distance limits quantity of water use. Similarly, the toilet facility is clean and in slightly good condition but shared by staff and patients; hand wash station present with water but no soap. The waste dumpster is present but overflowing and inside the PHC is clean but there are overgrown bushes outside. PHCs classified as No Service do not have water sources on premises, water is collected from water sources (ground or surface) more than 500 m. Water availability is influenced by yield and season. Toilet facility is in bad condition/No toilet facility. Hand wash station is absent, waste is disposed openly, and environment is dirty.

Data for this study was collected from secondary and primary sources. Data from secondary sources were collected from documents such as government publications (records of PHCs) and earlier research, while data from primary sources were collected through observation (participant) and interviewing (unstructured). Observation and interview method of primary data collection were considered ideal, as grading the sub-indicators required on-the-spot assessment of the WASH components of PHC facility and interviewing of facility staff where necessary. The choice of the unstructured method of interview allowed quality interactions between researchers and facility staff and enabled the collection of in-depth information.

### 2.2 Water quality testing of PHC water sources

Water samples were collected at all PHCs assessed across the states, to establish both the quality of service and accessibility. Fewer PHCs recorded no water sources while the types of water sources were detailed in Table S1 of the Supplementary file. *In-situ* physicochemical analysis was carried out to determine the pH, Electrical conductivity (E.C) and the Total Dissolved Solids (TDS) using PCR: 500 combo pH meter. The pH meter was calibrated using buffer 7 and deionized water. Other chemical parameters tested were Total Hardness (mg/L), Bicarbonate (HCO_3_^−^), Calcium (Ca^+^), Magnesium (Mg^+^), Chloride (Cl^−^), Nitrate - NO_3_^−^, Potassium (K^+^), Sulphate (SO_4_^−^) and *Escherichia coliform (E-coli)*. All water samples for the physicochemical testing were collected in 2 L Polypropylene bottles, carefully prerinsed with deionized water and the raw water samples, before collection. All testing were performed in triplicate for quality control and quality assurance purposes, following APHA (2017) standard procedure. The corresponding equipment and standard reagents for the water quality analysis were detailed in Ref. [34]. For example, bicarbonate was analysed using titrimetric method (0.02N of H_2_SO_4_), chloride was determined through addition of 1 ml of K_2_CrO_7_ in the samples before titrating against 0.01M of AgNO_3_.

## 3 Results and discussion

### 3.1 Indicator classification

**Fig. 3:**
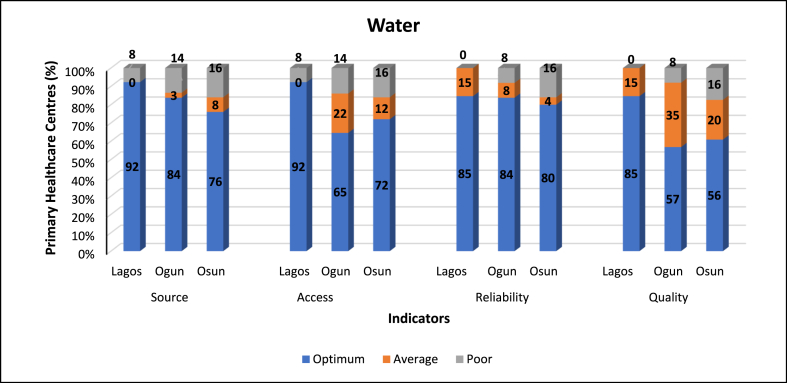
Indicators of water component of the WASH index.

**Fig. 4:**
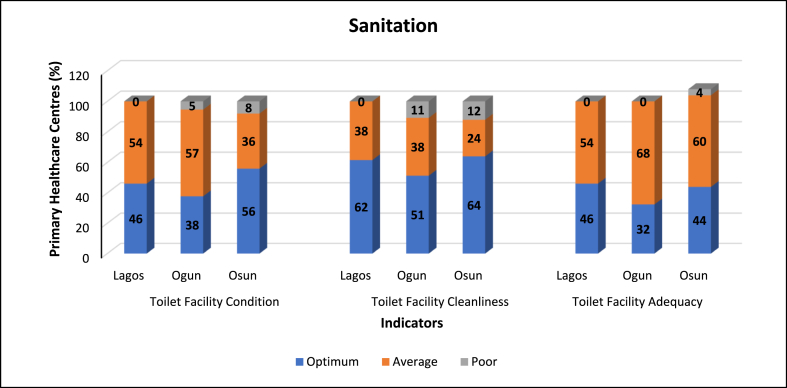
Sanitation component indicators of the WASH index.

**Fig. 5:**
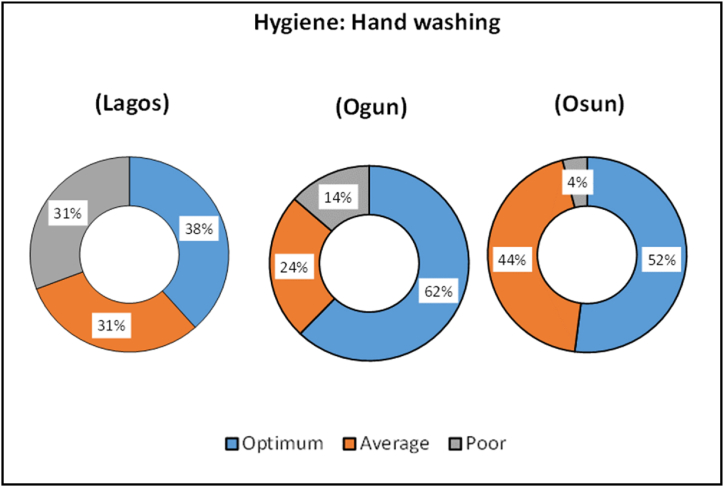
Indicators of hygiene component of the WASH index.

**Fig. 6:**
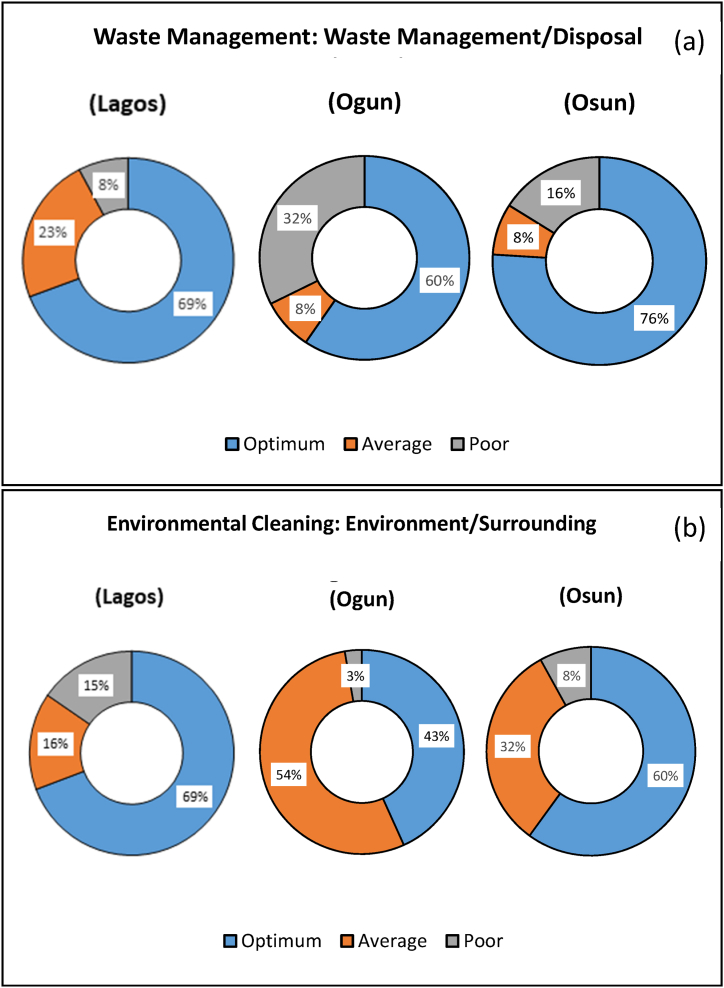
Indicators of (a) Waste management and (b) Environmental Cleaning Component Indicators of the WASH Index.

**Fig. 7:**
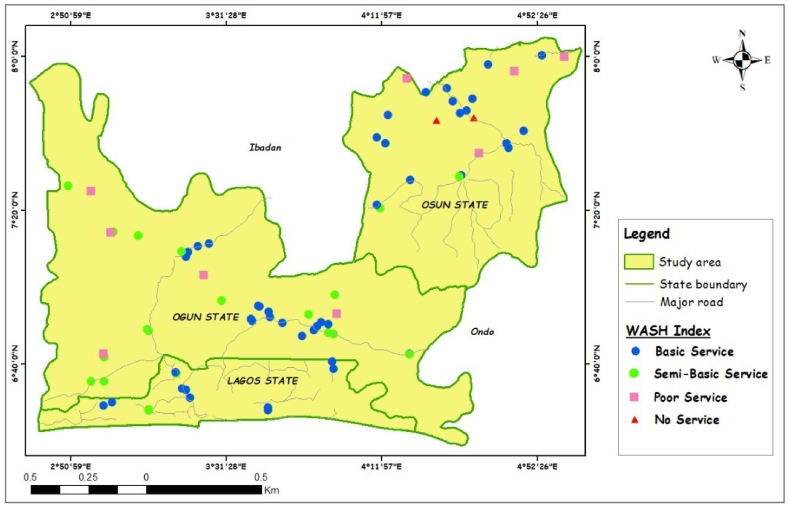
WASH index classification in the study area.

The results of the indicators of the WASH Index components are presented in Fig. 3, Fig. 4, Fig. 5, Fig. 6, Fig. 7. Fig. 3 indicates the graph of indicators of Water component of the WASH index. More PHCs scored Optimum [1] than Average [33] and Poor (0) for all indicators of Water component. Lagos PHCs had the highest indicator scores for all indicators of the water component, signifying that the water sources were located on premises, improved, with building connection, reliable and protected.

Access, reliability and quality indicators recorded significant percentage of PHCs with Average [33] score; Lagos PHCs (0 %, 15 % and 15 % respectively), Ogun State PHCs (22 %, 35 % and 35 % respectively) and Osun PHCs (12 %, 4 % and 20 % respectively).

Lagos PHCs appear to fair better with regards to all indicators of the Water component. The strategic position of Lagos State as a commercial nerve centre comprising of increased urban areas may be responsible for the quality of service in Lagos PHCs [[35], [36], [37]]. That does not undermine the efforts of the Ogun and Osun States governments in improving the state of PHCs in their respective States. The presence of safe and adequate water in PHCs is essential in the provision of quality service [33,16]. The provision of adequate water is regarded as one of the Minimum Standards for Primary Health Care in Nigeria regards provision of adequate water [38].

Sanitation indicators describe the state toilet facilities in the PHCs based on three [2] sub-indicators each according to Toilet facility condition, cleanliness, and adequacy (Fig. 4). Osun had the highest percentage of PHCs with Optimum scores for Toilet facility condition (56 %) and Toilet facility cleanliness (64 %), while Lagos and Osun PHCs had Optimum scores for Toilet facility cleanliness (62 % and 64 % respectively). Lagos PHCs scored the highest in Toilet facility adequacy (46 %). Generally, toilet facility type covers the technology type and cleanliness (for instance flush type/pit latrine), while toilet facility cleanliness and state of structure describes level of cleanliness and the state of the toilet facility structure. Toilet facility Adequacy is defined in terms of the number and condition of toilet facility/facilities in the PHC. The recommended minimum standard for sanitation (toilet facilities) advocated by Ref. [32] is categorized into 3: quantity, access and quality. For quantity, one toilet for every 20 persons in inpatient settings, 4 toilets in outpatient settings and separate toilets for staff and patients. With regards to access, availability of on-site toilet facility is the minimum standard. Lastly, the minimum standard for quality is a toilet facility that accommodates the local technical and financial conditions, is safe, clean, accessible to all users especially persons with reduced mobility.

Ogun PHCs had the highest Optimum scores (62 %) for Hygiene component (Fig. 5). Optimum scores for Hygiene (hand washing) indicates that the PHCs have functional hand washing stations, especially at point of care. It was observed that hand wash stations were present at the entrance and points of care for many PHCs, in compliance with COVID regulations.

The presence of adequate hygiene facilities at a time when the fight against COVID-19 is on, cannot be over-emphasized. Improved hand hygiene is described as the most cost-effective and practice means of in preventing the spread in infections in health facilities and antimicrobial resistance [39,40]. Infections can spread through care of multiple patient or from visitors to the PHCs [16]. WHO (2019) reports favours water and soap over alcohol-based hand rub particularly when hands are extremely dirty and after visiting the toilet.

Osun and Lagos PHCs were, however, better off for Waste Management (Fig. 6a), each with 76 % and 69 % Optimum scores respectively and Ogun following closely behind with 60 %. The Optimum score shows that waste was properly managed and disposed in over 60 % of the PHCs visited in the study area. This indicates an above average waste management and disposal performance with room for extensive improvement. Medical wastes are regarded as potentially non-hazardous due to their types and diverse sources, however, only 85 % are considered as hazardous [41]. Nonetheless, medical wastes are detrimental to humans and the environment and require proper handling/disposal to hazards associated with poor waste management [41,42].

In Fig. 6b, Lagos PHCs had the highest ‘Optimum’ score for environmental cleaning, followed by Ogun (43 %) and Osun (38 %). Optimum scores implies that the interior and exterior of the PHCs are kept clean and tidy. Grasses (if any) are cut and floors are kept clean with the use of disinfectants. Several key elements for prevent of infections in healthcare facilities including environmental cleanliness [43]. Environments in healthcare facilities are predisposed to harbour pathogens, putting patients at risk of multidrug-resistant organisms (MDRO) especially from previous patients [[43], [44], [45]]. Contaminated environmental surfaces facilitate the transmission of infection. Environment (of care/surfaces) in healthcare facilities refer to spaces where direct or indirect contact between a patient or healthcare facility staff occur and furniture and other fixed items in the facility [33,45]. There is limited information on the contributions of the environment of healthcare facilities to the risk of healthcare acquired infections, a situation may be attributed in part to poor funding of healthcare service delivery that impacts negatively on clean and safe environment of healthcare facilities [[45], [46], [47]].

### 3.2 WASH index

The WASH index classification determined from Equation (1) above shows that Lagos has the most PHCs in the Basic Service category (77 %), as shown in Fig. 7, followed by Osun (68 %) and Ogun (46 %). Ogun had the highest PHCs in the Semi-Basic Category (41 %), while Lagos and Osun scored 23 % and 8 % respectively.

Lagos did not record any PHC in the Poor Service. Osun had the highest percentage (16 %) of PHCs that recorded Poor Service. Only Osun had PHCs in the No Service category (8 %). Although 8 % is quite a small number, this does not negate the need for improvement in WASH facilities. Lagos State recording the highest number of PHCs in the ‘Basic Service’ category may be attributed to the status of the State as Nigeria's commercial nerve centre and the level of economic growth. This does not in any way underscore the efforts of the governments of Ogun and Osun States in providing quality healthcare for their residents. In addition, the absence of any PHC in Lagos and Ogun States in the ‘No Service’ category is a positive development, however, with room for further improvement. It is importance that government at all levels, pay critical attention to the State of primary healthcare. Nigeria currently ranks as one of the countries with the lowest healthcare outcomes globally, due to the poor services at many of her PHCs [[48], [49], [50]]. The failure of some of these PHCs to adequately deliver proper services has placed increased pressure on secondary and tertiary healthcare facilities. This comes at a huge cost (sometimes fatal), particularly due to proximity, poor road networks and finance.

As Nigeria struggles in the wake of her economic challenges, amidst the quest to reach her SDGs targets, there is no better time for the improvement in her primary healthcare system. The bedrock of economic growth and development of any country is premised on the development of human capital via adequate, sound and quality healthcare system [51]. Despite, Nigeria's oil wealth, the country healthcare spending per gross domestic product (GDP) is the second lowest in the world [50]; Nigeria's readiness in the provision of healthcare leaves more questions begging for answers.

### 3.3 Water quality at PHCs

Aside from the exposure to pathogens through WASH facilities hardware in PHCs, one major pathway is through the water quality, as water remains a key connector of the facilities (toilets, handwashing basins, waste management facility maintenance). The quality of water quality provided at individual centres could easily expose users (patients) to diverse contaminants such as coliforms, nitrate, among others. In this study, the quality of water from the water sources at the individual PHCs were assessed. Table 4 presents the water quality testing result of water sources across PHCs in Lagos, Ogun and Osun States, mean pH value were, 5.03 ± 0.89, 5.38 ± 1.26, and 6.39 ± 0.56, respectively. Lagos recorded the highest number of PHC water sources below the lower band of the World Health Organization (2017) acceptable water quality standard (pH < 6.5–8.5). The number of PHCs below the limit decreased from Ogun [15] and the lowest was recorded at Osun State [11], respectively. All pH values of the water samples in Lagos ranged from acidic, but two samples were acidic in Ogun, while 16 of the 25 water samples in Osun were acidic. Most of the water sources across the States are from groundwater exploitation (borehole and hand-dug wells), hence the acidic pH is considered as the product of general groundwater redox interactions that often influence pH. The pH of groundwater is naturally influenced by the dissolution of CO_2_ from rainwater and runoff in the aquifer, which in-turn affect the rate of neutralizing alkaline materials, metals mobility and rock – water interactive [34,52]. Additionally, the poor capability of the groundwater sources to neutralize the acidity is made obvious through the extremely low bicarbonate content in the water across the states. Bicarbonate (HCO_3_^−^) concentration ranges from Lagos (2.0 ± 1.04 mg/L), Ogun (1.89 ± 1.45 mg/L) and Osun 2.79 ± 1.75). Carbonate mineral dissolution process is one of the pathways of alkalinity in groundwater. Therefore, PHCs that utilize groundwater with these water sources are strongly recommended to adjust their water pH before usage, in an instance where treatment of the water source is too expensive.

**Table 4:** Summary of quality testing of water sources at PHCs across the States.

Parameter	Lagos State		Ogun State		Osun State		
	Mean ± S. D	No. outside Standard	Mean ± S. D	No. outside Standard	Mean ± S. D	No. outside Standard	WHO, (2017)
pH	5.03 ± 0.86	12	5.39 ± 1.24	31	6.39 ± 0.56	16	6.5–8.5
E. C (μS/cm)	540.15 ± 434	2	255.51 ± 244	0	364.14 ± 354	2	1000
TDS (mg/L)	270.42 ± 216	2	127.81 ± 122	0	149.66 ± 127	1	500
Total Hardness (mg/L)	116.77 ± 108	6	103.03 ± 114	27	242.26 ± 125	21	100
HCO_3_^−^ (mg/L)	2.0 ± 1.04	0	1.89 ± 1.45	0	2.79 ± 1.75	0	200
Ca (mg/L)	73.92 ± 66.56	1	52.68 ± 51.04	1	66.84 ± 53.07	1	200
Mg (mg/L)	42.85 ± 54	1	206.72 ± 211	17	175.74 ± 118	13	150
Cl (mg/L)	69.38 ± 47.10	0	40.62 ± 30.57	0	40.42 ± 28.3	0	250
NO_3_^−^ (mg/L)	6.04 ± 9.49	0	2.74 ± 3.5	0	1.18 ± 3.83	0	50
K (mg/L)	8.48 ± 10.39	2	3.97 ± 5.87	1	3.10 ± 6.14	1	<20
SO_4_^−^ (mg/L)	0.47 ± 0.64	0	2.22 ± 4.35	0	1.92 ± 8.63	0	250
*E. coli* (cfu × 10^3^)	4.27 ± 1.49	13	3.27 ± 3.0	30	3.84 ± 2.58	25	0 cfu

NB: The number of PHC presented for pH across the states are below the lower boundary of the WHO (2017) standard (pH = 6.5). No. above Standard (the total number of water samples per State exceeding the WHO maximum permissible limit). CFU = Coliform Unit.

The mean E.C and TDS were Lagos (540.15 ± 434 μS/cm and 270.42 ± 216), Ogun (255.51 ± 244 μS/cm and 127.81 ± 122 mg/L) and Osun (364.14 ± 354 μS/cm and 149.66 ± 127 mg/L), respectively. All water samples across the States are within established E.C and TDS limit of 1000 μS/cm and 500 mg/L, respectively. TDS is made up of inorganic salts and organic substances that make aesthetic alterations to water quality by creating poor taste and colour [53,54]. Similarly, Total Hardness of the water samples were below 100 mg/L, except for Six [5] sources in Lagos, 27 sources in Ogun and 21 sources in Osun. This shows that water sources at the PHCs were hard, which could either be through calcium hardness or magnesium hardness. Although the ionic classification was not carried in this study due to limited water quality parameters, the concentration of calcium and magnesium presented in Table 4 also confirmed that the water sources have a combination of calcium and magnesium hardness, with values of Lagos (73.92 ± 66.56 mg/L and 42.85 ± 54 mg/L), Ogun (52.68 ± 51.04 mg/L and 206.72 ± 211 mg/L) and Osun (66.84 ± 53.07 mg/L and 175.74 ± 118 mg/L), respectively. The number of PHC water sources above the WHO permissible limit of 150 mg/L for magnesium was highest at Ogun (17 PHC), followed by Osun [12], while Lagos recorded the least (1 PHC). Considering that the water usage is often for hand-washing and general cleaning, and probable consumption, ingestion of such water by users (patience and staffs) may cause mild to chronic stomach upset among other health implications, aside from the formation of scale on cooking/boiling utilities. This also implies that metal-based medical equipment such as scissors, etc., may easily corrode when sterilized with such water, posing more economic strain on the poorly funded PHCs.

All chloride and nitrate values did not exceed the WHO maximum permissible limits of 250 mg/L and 50 mg/L, respectively. The range of values for chloride for Lagos, Ogun and Osun States were 16–179 mg/L, 13–149 mg/L and 15–145 mg/L, respectively, while those for nitrate were 0–38.81 mg/L, 0.03–16.81 mg/L and 0–20.02 mg/L, respectively. Chloride occur in water as mineral solvents, capable of creating detectable taste and could pose some health effects in humans such as tooth decay, when consumed in high amounts [55]. Consumption of water with excess nitrate concentration is detrimental to human health and could cause methemoglobinemia, cyanosis (blue baby syndrome), thyroid hypertrophy and diverse kind of cancer [[56], [57], [58], [59], [60]]. Nitrate contamination in groundwater has been linked to agricultural waste, animal waste, poor wastewater management and leachate from solid wastes [58,61]. These have attracted research works on the health risks of nitrate and the correlation relationship between Nitrate and Chloride, as it provides insights on probable pollution from animal or human waste [34,57].

**Fig. 8:**
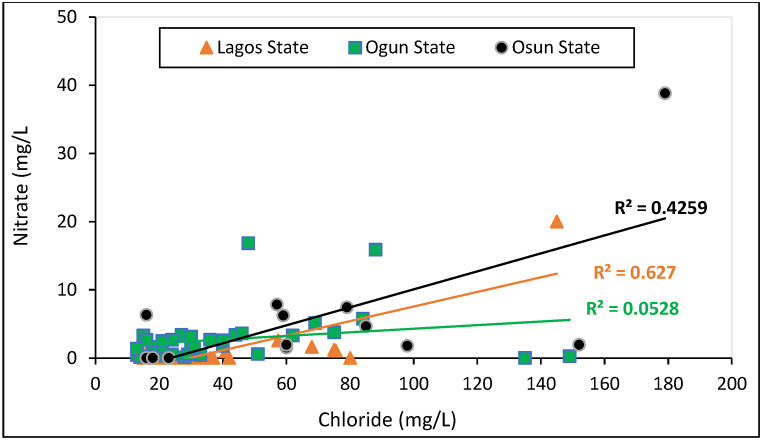
Correlation plot of Chloride vs. Nitrate for PHCs in Lagos, Ogun and Osun States.

Fig. 8 shows the correlation between nitrate and chloride in water sources across the States. The correlation plot was adopted to establish probable pollution from human and animal waste, septic tank seepage, oxidation of nitrogenous human and animal excreta, among others, as the source of nitrate and chloride association in the semi-arid region of India [57]. Although the nitrate concentration is generally low in the water samples (as shown in Table 4), high R^2^ value (0.627) in Lagos and slightly weak correlation in Osun (R^2^ = 0.426) suggests that some PHC water sources are vulnerable to diverse pollution sources including the human and animal excreta contamination. Ideally, in a rural setting, and urban PHCs where access to sanitation facilities is limited, patients and their relatives are likely to source for alternative means to pass out nitrogenous waste, particularly faeces and urine, which is made of over 50 % of Nitrogen [62]. Therefore, the seepage of such environmental pollution through which the nitrification process would likely be favored, is expected over a given period of time – enhanced by runoff. Thereby posing threats to the available water sources at the PHCs. The strong correlation in Lagos is understandable, considering the recent assertion of [63,64] that Lagos State has a high proportion of informal settlements where basic WASH facilities are heightened, leading to high rate of open defecation in Lagos. Additionally, studies have established that human excreta contributes up to 80 % of Nitrogen receives at urban municipal wastewater treatment facilities across the globe [62]. Hence, human excrete could be a probable pathway for nitrate-chloride associated contamination across the PHCs, among other sources.

The low correlation score recorded at Ogun State (R^2^ = 0.0528) shows that nitrogen and chloride pollution across the PHCs are not related, particularly to fecal and nitrogen related sources such as seepage from septic tanks, improper wastewater, and solid waste handling, among others. Also, considering the nitrate concentration recorded in Ogun and Osun State, compared to Lagos State (Table 4), it could be established that diverse nitrogen pollution sources that are associated with chloride influenced the high ratio recorded in Lagos, despite the state recording the overall best WASH index score. This further emphasized that water quality evaluation could reinforce the findings from qualitative service evaluation such as the WASH index and JMP service ladder, among other indices. Additionally, the lithology of Lagos could also influence the transport of these pollutants from nearby surrounding of the PHC, while we must also understand that these are beyond the jurisdiction of the PHC staff/authority. Generally, the pollutant concentration is currently low compared to the WHO standard. Nonetheless, Fig. 8 established that general environmental cleaning is essential to be pursued in Lagos State and Osun State to curb future escalation of the pollution level.

Potassium concentrations across the States were Lagos (8.48 ± 10.39 mg/L), Ogun (3.97 ± 5.87 mg/L) and Osun (3.10 ± 6.14 mg/L), with the number of PHC water sources above the 20 mg/L limit by WHO (2017) ranged as presented in Table 4. Meanwhile, all water sources across the States recorded sulphate concentration below the 250 mg/L stipulated standard by WHO (2017). Thirty [29] PHC water sources tested from Ogun State recorded the presence of *E. coli*. Followed by twenty-four (out of 25) PHC water sources in Osun, recorded the presence of *E. coli*. While all PHC water sources in Lagos had *E. coli* contamination. WHO recommends that no form for bacteria colony should be present in drinking water – zero coliform. Notably, Lagos has 100 % of PHCs water sources with coliform count, which presents a serious public health concern as users are highly susceptible to varying degrees of illness, particularly, diarrhoea, vomiting and dysentery. The detection of *E. coli* in water samples is a concern as the water does not undergo any treatment before use (particularly for hand washing). This puts the health of patients and staff of the respective PHCs at risk. The bacterial contamination in water sources further strengthen the assumption of probable human and animal excreta as a major source of pollution across the PHCs, in addition to the septic tank seepage, mishandling of sanitary and solid wastes in the environment.

Generally, our findings have established through the water quality testing of water sources at PHCs that access to facilities is as important as the quality of the water used in WASH facilities, considering the crucial role of water in WASH. For instance, despite Lagos recording the highest number of Basic WASH service across over 90 % of their PHCs, water sources across these same PHCs are considered unsafe for use (based on the *E-coli* results). This presents a reason for holistic evaluation of WASH services, beyond the physical evaluation that is usually done with indices. Similarly, the pH results across the PHCs with most sources having slightly acidic pH shows that users who are pregnant may be at more risks. This is because the pregnant women are envisaged to frequent PHCs more than other patience due to their pregnancy (antenatal), meanwhile, acidic pH enhances metals mobility in water [34,65]. Additionally, most water sources across the PHCs are from groundwater, thus, these water sources may likely have metal contamination since most rocks are rich in metals such as cadmium, iron, zinc, among others [34,66,67]. Whereas, exposure to iron, zinc and copper poses great threat on pregnancy, causing severe health conditions for both the mother and foetus, such as low birth weight, placental insufficiency, pre-eclampsia, pregnancy induced hypertension, teratogenic, chaemochromatosis, etc. [68]. These affirms that adequate monitoring is essential to guarantee the safety of PHC users based on the WASH results, futher facilitating the realization of WASH security in PHCs. Therefore, it becomes imperative to carefully sensitize both PHCs operators and users on water quality management and environmental management practices, to further enhance the effectiveness of appropriate hand washing behaviour at PHCs.

### 3.4 Local realities and the need for WASH security

Challenges facing the health sector in Nigeria are multifaceted. Addressing Nigeria's health challenges requires government and societal approach in their totality [48]. This involves cost effective health-promoting policies and interventions investments, with high cost-benefit ratios. At the Organisation of African Unity (now African Union) Abuja Declaration of April 2001, government across pledged to allocate 15 % of their budget for the health sector. However, budgetary allocation in the health sector is still inadequate [69] with allocations below 15 %.

Despite underfunding, corruption in the health sector is another factor. Corruption manifests in many dimensions through weak governance, accountability, poor remuneration of health care staff, lack of transparency and weakly enforced procurement laws [[70], [71], [72], [73]]. The outcome is dilapidated and improperly maintained infrastructure, ineffective service delivery resulting poor customer satisfaction, and, in some cases, fatalities could occur where there is total breakdown of service and alternative health care facilities are far away.

Studies suggested vertical and horizontal approaches to addressing corruption in the health sector [72,[31], [74], [75]]. Vertical approaches consist of creation of accountability through appropriate rules/regulations, provision of incentives for compliance with regulations and implementation of appropriate sanctions. Horizontal approaches comprise agreements between relevant stakeholders such as health workers and managers. Although the level of effectiveness of these approaches has been reported to be low [72,74,75]. The enduring corruption in the health sector is enhanced in part by inadequate enforcement of penalties on defaulting officers and service providers and this may in turn frustrate responses to demands for efficient service delivery.

Notwithstanding the corruption challenges and as efforts are made to effectively address the challenges, prioritizing WASH is key. Ensuring the provision of adequate and sustainable WASH facilities across all health facilities is important in achieving WASH security, especially as the world is yet to recover from the effect of COVID-19 pandemic. WASH security could be achieved through, 1) provision of adequate, functional, and properly maintained WASH facilities and 2) the awareness of patients/visitors to health care facilities on their right to appropriate WASH facilities and the need to demand such where not found. The downside to the latter, however, is the possible absence of appropriate channels to seek redress when access to WASH facilities is denied or favourable response when complaints are made. For instance, relatives of women who give birth in PHCs without water supply have been reported to source for water on their own for clean-up [76]. Expectedly, such women may have to subsequently keep complying with bringing water to the PHC or seeking medical intervention at a farther health care facility (which may not come cheap and without challenges regarding logistics). If the conditions of some health facilities have deteriorated to the level of patients bringing water for personal use, sanitation and hygiene status of such facilities are better imagined. Emphasis on WASH security for patients/visitors and staff of health care facilities is therefore paramount. It is important to mention that WASH Security is not limited to PHCs as households, workplaces and schools are also important for a sustainable society to be achieved.

## 4 Conclusion

The status of WASH in PHCs in selected States in Southwest Nigeria was accessed using research driven WASH Index in this study. The WASH Index has provided a composite assessment tool for assessing the status of WASH in health care facilities. Lagos recorded the most PHCs in the Basic Service category (77 %), Ogun had the highest PHCs in the Semi-Basic Category (41 %), Osun had the highest percentage (16 %) of PHCs that recorded Poor Service and Osun State was the only State that recorded PHCs with ‘No Service’. It was interesting to note that Lagos did not record any PHC in the ‘Poor Service and ‘No Service’ category. This finding depicts the influence of economy and the metropolitan status of Lagos as a city and by extension as a State on the quality of health services provided especially at the level of primary healthcare. This, however, does not undermine the efforts of the governments of the other two States.

The concise nature of the WASH index and ease of computation could be an advantage to data collection when applied to WASH related studies. PHCs, particularly in Poor and No Service categories are worrisome, with the goal of minimum, affordable and proximal health care that PHCs are expected to provide jeopardized, not thanks in part, to underfunding and corruption. It is believed that sub-optimal service has endured till date due to the lack of awareness that access functional health care service is a basic right of patients and patients’ refusal or inability to demand quality service. Provision of quality service remains the responsibility of the government.

The importance of WASH security has also been highlighted. Ensuring patients and staff at health care facilities are WASH secure is important to prevent the spread healthcare acquired infections. WASH insecurity is enhanced by inadequate and unsustainable WASH facilities, which was identified in some PHCs. For instance, PHCs without water supply increases the risk infections and has direction impact on sanitation and hygiene components of WASH. Average toilet facility condition and toilet facility adequacy recorded in this study is a concern. Besides water supply challenges, toilet facility condition and toilet facility adequacy are critical as poor and inadequate toilet facility can encourage open defecation; a development that would impact the race towards the Sustainable Development Goals. Functional hand hygiene facilities and improved waste management and environmental cleaning practices are also necessary for quality healthcare service delivery.

Future studies should explore the implementation of the index at secondary and tertiary healthcare facility levels, to ensure appropriate service monitoring and evaluation, and WASH security at all scales of the healthcare systems. Adoption of the proposed index would facilitate holistic WASH monitoring concept (qualitative and quantitative) particularly in the global south.

## 5 Limitations of study

Authors encountered three major challenges including accessibility, insecurity and funding. Some PHCs were located in areas that were difficult to reach due in part to poor road network and the insecurity in some parts of the study area influenced the coverage of the study. Funding was limited as the study was carried out using authors’ personal funds. It is expected that the study would be carried out on a larger scale to cover the rest of the Southwest region of Nigeria in external funding is obtained.

### Ethical approval

Ethical approval waiver was obtained for this study in accordance with the research ethics criteria of the National Code of Health Research Ethics, Federal Ministry of Health, Nigeria.

## Funding

The study is a pilot study to test the WASH Index tool and carried out using authors’ personal funds.

## Data availability statement

Authors declare that all data associated with this study has been presented in the manuscript and data are available upon request.

## CRediT authorship contribution statement

**Enovwo E. Odjegba:** Writing – review & editing, Writing – original draft, Visualization, Supervision, Resources, Project administration, Methodology, Investigation, Formal analysis, Data curation, Conceptualization. **Abayomi O. Bankole:** Writing – review & editing, Visualization, Supervision, Resources, Methodology, Investigation, Formal analysis, Data curation, Conceptualization. **Adebayo Sadiq:** Visualization, Supervision, Methodology, Investigation, Data curation, Conceptualization. **Barakat O. Layi-Adigun:** Writing – review & editing, Supervision, Methodology, Investigation, Conceptualization. **Abayomi M. Adebimpe:** Visualization, Validation, Formal analysis, Data curation. **Mariam O. Kosemani:** Visualization, Validation, Formal analysis, Data curation. **Emmanuel B. Ojo:** Visualization, Validation, Formal analysis, Data curation. **Mustapha A. Adewuyi:** Visualization, Validation, Project administration, Formal analysis, Data curation.

## Declaration of competing interest

The authors declare that they have no known competing financial interests or personal relationships that could have appeared to influence the work reported in this paper.
