# Supplementary material for: Water, sanitation and hygiene (WASH) index for primary healthcare facilities: Towards achieving WASH security
Source: Heliyon. 2024 Aug 2;10(15):e35548. doi: 10.1016/j.heliyon.2024.e35548 (PMC11334800; doi:10.1016/j.heliyon.2024.e35548)
Supplement: Multimedia component 1 [file mmc1.docx]

Supplementary file.

Table S.1: Summary of PHC Water Source Type across Lagos, Ogun and Osun States, Nigeria.

| **SN** | **Name of PHC** | **LGA** | **Source Type** | **Water Source** |
| --- | --- | --- | --- | --- |
|  | **Lagos State** |  |  |  |
| 1 | Ogunlana PHC | Oshodi-Isolo | Borehole | Protected |
| 2 | Ajibulu PHC | Oshodi-Isolo | Borehole | Protected |
| 3 | Odunbaku PHC | Orile Agege | Borehole | Protected |
| 4 | Powerline PHC | Orile Agege | Borehole | Protected |
| 5 | Ilogbo-Eremi PHC | Badagry | Borehole | Protected |
| 6 | Isalemo PHC | Badagry | Borehole | Protected |
| 7 | Okokomaiko PHC | Ojo | Hand-dug well | Semi Protected |
| 8 | Kemberi PHC | Ojo | Hand-dug well | Unprotected |
| 9 | Eredo PHC | Epe | Borehole | Protected |
| 10 | Mojoda PHC | Epe | Borehole | Protected |
| 11 | Awoyaya PHC | Ibeju Lekki | Borehole | Protected |
| 12 | T&G PHC | Ibeju Lekki | Hand-dug well | Semi Protected |
| 13 | Oba Salami PHC | Lagos Mainland | Borehole | Protected |
|  | Ogun State |  |  |  |
| 1 | Imeko PHC | Imeko | Borehole | Protected |
| 2 | Afon PHC | Imeko | River | Unprotected |
| 3 | Idofoi PHC | Yewa North | Borehole | Protected |
| 4 | Family health care center Ibile Ayetoro | Yewa North | Hand-dug well | Semi Protected |
| 5 | Kuto PHC | Abeokuta South | Borehole | Protected |
| 6 | Ijaye PHC | Abeokuta South | Borehole | Protected |
| 7 | Olorunda PHC | Abeokuta North | Borehole | Protected |
| 8 | Enugada PHC | Abeokuta North | Borehole | Protected |
| 9 | Obantoko PHC | Odeda | Borehole | Protected |
| 10 | Osiele PHC | Odeda | Borehole | Protected |
| 11 | Papalanto PHC | Ewekoro | Borehole | Protected |
| 12 | Olose PHC | Ifo | Borehole | Protected |
| 13 | Oke-Nla PHC | Ifo | Borehole | Protected |
| 14 | Ado-Odo PHC1 | Ado-Odo/Ota | Borehole | Protected |
| 15 | Ado-Odo PHC2 | Ado-Odo/Ota | Borehole | Protected |
| 16 | Alagbon PHC | Yewa South | Borehole | Protected |
| 17 | Isaga PHC | Yewa South | Public Water Supply | Protected |
| 18 | Isoku PHC | Ijebu-Ode | Borehole | Protected |
| 19 | Italapo PHC | Ijebu-Ode | Borehole | Protected |
| 20 | Atan PHC | Ijebu Northeast | Hand-dug well | Unprotected |
| 21 | Eruwon PHC | Ijebu Northeast | Borehole | Protected |
| 22 | Oke-Ago PHC | Ijebu North | Borehole | Protected |
| 23 | Obada | Ijebu North | Borehole | Protected |
| 24 | Ilese PHC | Ijebu East | Borehole | Protected |
| 25 | Imushin PHc | Ijebu East | Borehole | Protected |
| 26 | Fegon Memorial | Ogun Waterside | Borehole | Protected |
| 27 | Orita J4 | Ogun Waterside | Borehole | Protected |
| 28 | PHC Owode | Obafemi Owode | Borehole | Protected |
| 29 | Kobape Health Post | Obafemi Owode | No water source | Unprotected |
| 30 | Makun PHC | Shagamu | Borehole | Protected |
| 31 | Ewu Oliwo PHC | Shagamu | Borehole | Protected |
| 32 | Ultra-modern Odogbolu PHC | Odogbolu-Ikene | Borehole | Protected |
| 33 | Alatise Clinic | Odogbolu-Ikene | Borehole | Protected |
| 34 | Remo PHC | Remo | Hand-dug well | Protected |
| 35 | Iperu-Remo PHC | Remo | Borehole | Protected |
| 36 | Ikene Health Clinic | Ikene | Borehole/Rainwater Harvesting | Protected |
| 37 | Healing Spring | Ikene | Public Water Supply | Protected |
|  | Osun State |  |  |  |
| 1 | Ogunlade PHC | Ila Orangun | Borehole | Protected |
| 2 | Alagbede PHC | Ifedayo | River | Nil |
| 3 | Comprehensive PHC | Boluwaduro | Borehole | Protected |
| 4 | Emiloju PHC | Ifelodun | Borehole | Protected |
| 5 | Ife Central PHC | Ife Central | Borehole | Semi Protected |
| 6 | Akarabata PHC | Ife East | Hand-dug well | Protected |
| 7 | Atakumosa PHC Osu | Ife West | Borehole | Protected |
| 8 | Bode Oshi PHC | Ola Olu | Borehole | Protected |
| 9 | Saka Saka PHC | Ejigbo | Hand-dug well | Protected |
| 10 | Ara PHC | Egbedore | Borehole | Protected |
| 11 | Aromiwe PHC | Irepodun | Hand-dug well | Protected |
| 12 | Eleesi PHC | Orolu | Borehole | Protected |
| 13 | Oke Baale PHC | Boripe | Borehole | Protected |
| 14 | Ota Efun PHC | Olorunda | Borehole | Protected |
| 15 | Ogo Oluwa PHC | Osogbo | Borehole | Protected |
| 16 | Irepodun PHC | Ede North | Borehole | Protected |
| 17 | Adeti PHC | Ilesa West Central | Borehole | Protected |
| 18 | Oriade PHC | Oriade | Borehole | Protected |
| 19 | Atakumosa PHC | Atakumosa East | No Source | Nil |
| 20 | Orojo PHC | Ilesa East | Borehole | Protected |
| 21 | Iwo PHC | Iwo | Borehole | Protected |
| 22 | Oke Ofa PHC | Isokan | Borehole | Protected |
| 23 | Ayedaade PHC Ikire | Irewole | Borehole | Protected |
| 24 | Akiriboto Oke PHC | Ayedaade | Borehole | Semi Protected |
| 25 | Oke Odo PHC | Ayedire | Borehole | Protected |
